# Harvard Odontological Society

**Published:** 1893-09

**Authors:** 

**Affiliations:** Harvard Odontological Society


### HARVARD ODONTOLOGICAL SOCIETY.

The regular meeting of the Harvard Odontological Society was held at Young's Hotel, Boston, April 27, 1893, President Eddy in the chair.

A paper was read by Henry P. Talbot, Ph.D. (Massachusetts Institute of Technology). Subject, "Some of the Properties of Hydrogen Peroxide Solutions."

(For Professor Talbot's paper, see page 646.)

### DISCUSSION.

*Dr. Taylor.*—One of the dental journals states that in making a solution of peroxide of sodium, cooling the water is not required, but simply that the powder to be dissolved be poured in very slowly. I would like to ask Professor Talbot about that.

*Professor Talbot.*—I do not know from practical experience just how far that can be done. In one case they say, cool the solution and pour it fairly rapidly, the object being to avoid heat. In the same way you might avoid heat by pouring it in slowly. Of course, just as soon as the decomposition begins it warms up the more, and the more it decomposes.

In connection with the sodium peroxide, you will find directions on the can to seal it after removing what is required, and I would strongly advise not taking any more than you need at the time, as, if it takes no moisture or carbonic acid from the air, the peroxide will gradually undergo decomposition, and after a little it is useless.

There is this difference between the peroxide of sodium and the peroxide of hydrogen: Sodium hydrate is a caustic alkali, and those solutions of peroxide which you make from it would be more or less caustic, whereas the hydrogen solutions would be acid. You will also find directions on the can to avoid grinding it. The peroxide of sodium is a substance which will liberate oxygen very rapidly, and if there is, by chance, organic matter present, the result may be something similar to that obtained when potassium chlorate and sulphur are ground together; the peroxide might give up its oxygen so rapidly as to cause a serious explosion.

*Dr. Page.*—Why could not these solutions be prepared and put in ounce vials, and then used as wanted, throwing away what is not used, and taking the next time a fresh vial?

*Professor Talbot.*—That could be done; but it would be better to let some one keep it for you. The fifty-volume solutions of peroxide of hydrogen are so placed on the market.

*Dr. Page.*—The tube that I use has a gold point about an inch and a half long, and is very small at the opening. Is there any harm in using such a point?

*Professor Talbot.*—If you leave your instrument in the solution for any length of time there would probably be decomposition. It is recommended that glass instruments be used.

*Dr. Upham.*—There are tubes made of gold and lined with platinum, very small and attenuated, for applying peroxide to the root-canals of teeth. I would like to ask Professor Talbot what he thinks of the value of such a tube,—would it be better to use that, or simply a gold tube?

*Professor Talbot.*—I should say the gold tube. Gold does not evolve oxygen so rapidly as platinum, and the chances of decomposition would be less; but one must remember that a very short contact with either gold or platinum would perhaps do a great deal of harm to the peroxide.

*Dr. Clapp.*—Would it make any difference if the remedy be neutralized?

*Professor Talbot.*—If it is brought just to the neutral point it would be just as efficacious for your purposes, though not quite so for an actual germicide. One would have to use care in attempting to neutralize it, for if it goes beyond the neutral point, there is a spontaneous and rapid decomposition.

*Dr. Clapp.*—Is there any change in the appearance at such a time?

*Professor Talbot.*—Yes; you see the oxygen coming off.

*Dr. Clapp.*—The reason I have used it so little is on account of its deteriorating so rapidly; and I also think that the acid in its composition has an injurious effect. Where I have used it in pockets around teeth, it seems to me to affect the roots, making them very sensitive.

*Dr. Stoddard.*—I took a tooth which was freshly extracted and left it in a fifty-volume solution for about thirty days, and it did not soften the enamel at all.

*Dr. Clifford.*—Will Dr. Eddy tell us his experience with peroxide of hydrogen?

*President Eddy.*—I have used peroxide of hydrogen for three years, buying it in pound bottles. I recommend it to patients as a mouth-wash, and it keeps the mouth in good condition. I have sometimes changed to listerine, which has much stronger acid properties than the peroxide, but is nevertheless an excellent mouth-wash.

*Dr. Clapp.*—What would be the best agent for following the hydrogen peroxide to get rid of the acid, water or alcohol?

*Professor Talbot.*—It would make very little difference; it is probably more soluble in water. What would probably be the effect of a caustic following an acid?

*Dr. Clapp.*—I should imagine there would be no perceptible effect on the tissue; we use caustic potash in connection with carbolic acid for pyorrhœa.

*Dr. Taylor.*—If the higher volumes of caustics are used, it is recommended that they be followed by a dilute acid.

*Dr. Stanton.*—I would like to ask Professor Talbot if he has investigated the properties of pyrozone? One of the professors of the College of Pharmacy called my attention to it.

*Professor Talbot.*—No, sir; I have not.

*President Eddy.*—It is claimed to have a strength of fifty volumes in ether. It is very caustic, and is manufactured by McKesson & Robbins, New York, who put out a caustic solution and an antiseptic. It comes in ounce bottles.

*Dr. Clifford.*—Do you use it?

*President Eddy.*—I have used pyrozone about a year. It is very good to remove stains from the necks of the teeth where the gum has receded.

*Dr. Bigelow.*—I would like to ask Professor Talbot if, when we have a solution of peroxide which we think may have lost its virtue as a germicide, we apply that to putrescent matter, and oxygen is liberated, that is evidence that it still has the germicidal power?

*Professor Talbot.*—Not necessarily, because the germicidal power seems to fall off before the evolution of gas ceases.

*Dr. Bigelow.*—Then really the only way we can tell is by such a test as you have made to-night?

*Professor Talbot.*—That is about the only way; there have been proposed various methods for quick testing, but I know of none of them that I could recommend.

*Dr. Boardman.*—I have used peroxide of hydrogen freely for two or three years, and have never had trouble with it in the way of disintegration. I use it with pumice in cleansing teeth.

*Dr. Bigelow.*—I have always supposed that as long as gas was generated it still possessed germicidal properties.

*Professor Talbot.*—I do not mean to say that it is entirely useless for your purposes, but you do not want to accept it as a prompt germicide simply because it evolves oxygen. In cavities, for instance, there is a greater chance for the action to take place than there would be on the diphtheritic membrane, because there it would be promptly diluted by the saliva. The commercial solutions might be satisfactory in many cases where it is used by you, but when you ask if it is a germicide, speaking in the sense in which that term is used in the biological laboratory, I think I should have to say, probably not.

*Dr. Bigelow.*—How long must it remain in contact with germs to destroy them?

*Professor Talbot.*—A fifty-volume solution would kill the diphtheria bacilli in twenty seconds.

*Dr. Taylor.*—What would be a good strength of potassium permanganate solution to use in a graduated test-tube?

*Professor Talbot.*—Five grammes to the litre of potassium permanganate.

*Dr. Taylor.*—The amount reduced by a given quantity of the peroxide tells the strength of solution?

*Professor Talbot.*—Yes, in the proportions about as I showed you this evening.

*Dr. Bigelow.*—Would the peroxide of hydrogen penetrate tooth-substance itself enough to act as a germicide?

*Professor Talbot.*—As it does not attack sound tissue readily, I do not think it would. That question is too purely physiological, however, for me to answer.

*Dr. Bigelow.*—What we try to do, and think we do in some cases, is to apply some agent which will saturate the dentine as thoroughly as the germs have.

*Professor Talbot.*—I should say that it would follow wherever tissue has become diseased. There would be some capillary attraction there.

*Dr. Werner.*—Of what volume is the solution that we generally obtain at the druggists?

*Professor Talbot.*—Probably between fifteen and eighteen volumes. Most of the commercial solutions are of about that strength.

HENRY L. UPHAM, D.M.D.,
*Editor Harvard Odontological Society.*

